# Quercetin Attenuates *Streptococcus pneumoniae*-Induced Oxidative Stress, Inflammatory Mediator Release, and Cytotoxicity in Human Bronchial Epithelial Cells: An In Vitro Study Using an Acapsular Strain

**DOI:** 10.3390/microorganisms14102140

**Published:** 2026-09-23

**Authors:** Mansoor Alsahag

**Affiliations:** Department of Clinical Laboratory Sciences, Faculty of Applied Medical Sciences, Najran University, P.O. Box 1988, Najran 61441, Saudi Arabia; maalsahag@nu.edu.sa

**Keywords:** quercetin, *Streptococcus pneumoniae*, oxidative stress, reactive oxygen species, catalase, superoxide dismutase, glutathione, IL-8, bronchial epithelial cells, in vitro

## Abstract

*Streptococcus pneumoniae* can induce airway epithelial oxidative stress, inflammatory mediator release, and cellular injury. Quercetin has reported antioxidant and anti-inflammatory properties, but its protective effects during pneumococcal epithelial challenge require cautious experimental validation, including direct ROS measurement, dose-response testing, viability confirmation, bacterial-load control, and comparison with a positive-control antioxidant. Human BEAS-2B bronchial epithelial cells were exposed to *S. pneumoniae* R6x at 10^6^ CFU/mL for 24 h with or without 2 h quercetin pretreatment. The main quercetin concentration was 20 µM, with additional dose-response testing at 5, 10, 20, and 40 µM. Oxidative stress was assessed by catalase activity, SOD activity, GSH/GSSG ratio, DCFH-DA fluorescence, and MitoSOX fluorescence. Inflammatory mediators were quantified by IL-8 ELISA and broader cytokine profiling. Cell injury and viability were assessed by LDH release and MTT assay. Bacterial burden was measured by CFU plating, and N-acetyl-L-cysteine was included as a positive-control antioxidant. *S. pneumoniae* reduced catalase activity, SOD activity, and GSH/GSSG ratio while increasing IL-8 secretion and LDH release. Quercetin restored catalase activity from 13.98 ± 1.51 to 25.77 ± 2.76 units/mg protein, restored SOD activity from 6.92 ± 1.02 to 12.46 ± 1.22 units/mg protein, and improved the GSH/GSSG ratio from 0.218 ± 0.013 to 0.486 ± 0.013. Quercetin reduced IL-8 from 11,444.49 ± 1476.92 to 3448.57 ± 247.52 pg/mL and LDH cytotoxicity from 44.61 ± 5.03% to 11.97 ± 2.46%. Direct ROS assays confirmed suppression of DCFH-DA-associated fluorescence and mitochondrial superoxide (MitoSOX) signal. Dose-response analysis supported 20 µM as an effective non-toxic concentration. MTT assay confirmed improved metabolic viability, while CFU assays indicated that quercetin did not substantially reduce bacterial burden under the tested conditions. Under the preventive conditions tested, involving 2 h of quercetin pretreatment before bacterial exposure, quercetin showed an epithelial-cell protective profile in BEAS-2B cells exposed to live acapsular *S. pneumoniae* R6x. These findings support a preventive cytoprotective effect of quercetin pretreatment in this in vitro model and require validation in primary airway epithelial cultures, encapsulated pneumococcal strains, post-infection treatment designs, antibiotic-combination settings, and in vivo pneumonia models.

## 1. Introduction

*Streptococcus pneumoniae* remains a major bacterial pathogen responsible for community-acquired pneumonia, meningitis, sepsis, and invasive pneumococcal disease. In the respiratory tract, pneumococcal disease reflects a complex interaction between bacterial virulence factors, epithelial barrier responses, innate immune activation, and host inflammatory injury [1,2]. Although antibiotic therapy and vaccination have substantially reduced disease burden, pneumococcal pneumonia continues to cause clinically important morbidity, particularly when infection is accompanied by excessive inflammation, epithelial damage, or progression to acute lung injury. Thus, adjunctive protective approaches that limit epithelial injury and inflammatory amplification, without replacing antimicrobial therapy, remain of mechanistic interest [3,4].

Airway epithelial cells are not passive targets during pneumococcal infection. They detect bacterial products, respond to oxidative and metabolic stress, and release cytokines and chemokines that shape downstream leukocyte recruitment [5]. Pneumococci can contribute directly to oxidative stress through bacterial hydrogen peroxide production and indirectly through virulence-factor-mediated host-cell stress responses [6,7,8]. Pneumolysin and pneumococcal autolysis have also been linked to epithelial injury, mitochondrial dysfunction, and release of mitochondrial danger-associated signals, suggesting that redox imbalance and mitochondrial perturbation are biologically relevant features of pneumococcal epithelial injury [9,10,11]. These studies support the rationale for evaluating both global intracellular ROS and mitochondrial ROS, rather than relying only on indirect antioxidant enzyme measurements [12].

The airway epithelial antioxidant system includes enzymatic defenses such as superoxide dismutase and catalase, and non-enzymatic thiol buffering systems such as reduced glutathione. Superoxide dismutase converts superoxide to hydrogen peroxide, catalase limits hydrogen peroxide accumulation, and the GSH/GSSG balance reflects intracellular redox reserve. Disruption of this antioxidant network can amplify oxidative injury and inflammatory signaling. Therefore, simultaneous assessment of catalase activity, SOD activity, the GSH/GSSG ratio, DCFH-DA fluorescence, and MitoSOX fluorescence provides a more integrated view of oxidative stress than any single readout alone. Nevertheless, fluorescent ROS probes have interpretive limitations; DCFH-DA and MitoSOX are useful screening tools but should be interpreted alongside complementary biochemical and viability assays rather than as fully specific quantitative measures of individual ROS species [13].

Quercetin is a dietary flavonoid with reported antioxidant and anti-inflammatory properties [14]. Its actions are context-dependent and may include direct redox activity, modulation of antioxidant-response pathways, and suppression of inflammatory signaling pathways such as NF-κB and MAPK in selected cell systems [15,16,17]. In airway epithelial cells, quercetin has been shown to reduce cytokine-induced chemokine expression, including IL-8/CXCL8 and CCL2/MCP-1, supporting its potential relevance to epithelial inflammatory responses [18].

This study therefore examined whether quercetin attenuates oxidative stress, inflammatory mediator release, and cytotoxicity in BEAS-2B bronchial epithelial cells exposed to the acapsular *S. pneumoniae* D39 derivative R6x. The study was designed as a mechanistic in vitro model rather than as a therapeutic efficacy study. To strengthen the interpretation and address common experimental limitations, the analysis included endogenous antioxidant readouts, direct total and mitochondrial ROS assays, cytokine/chemokine profiling, LDH and MTT viability assays, quercetin concentration-response testing, bacterial-load controls, and an N-acetyl-L-cysteine positive-control antioxidant group. We hypothesized that quercetin pretreatment would reduce pneumococcus-induced epithelial oxidative stress and inflammatory amplification without major suppression of bacterial burden under the tested conditions.

## 2. Materials and Methods

### 2.1. Cell Culture

Human bronchial epithelial BEAS-2B cells (ATCC, Manassas, VA, USA; CRL-9609) were cultured in BEGM Bronchial Epithelial Cell Growth Medium BulletKit, consisting of BEBM basal medium and SingleQuots supplements (Lonza, Walkersville, MD, USA; catalog no. CC-3170). Cells were maintained at 37 °C in a humidified 5% CO_2_ incubator and used at approximately 70–80% confluence. Cells were seeded in 96-well, 24-well, or 6-well plates according to the downstream endpoint. For infection experiments, cells were pretreated with quercetin for 2 h before exposure to *S. pneumoniae*.

### 2.2. Bacterial Strain and Culture

The bacterial strain was the unencapsulated D39 derivative R6x, derived from *S. pneumoniae* serotype 2 D39 (laboratory stock). Pneumococci were streaked on Columbia agar supplemented with sheep blood and incubated overnight at 37 °C with 5% CO_2_. Bacteria were grown in Todd–Hewitt broth supplemented with 5% yeast extract to mid-logarithmic phase (OD600 = 0.2–0.4). Suspensions were centrifuged at 1800× *g* for 10 min, washed, and resuspended in epithelial-cell culture medium. Bacterial concentration was determined by optical density with reference to a predetermined OD_600_-CFU calibration curve and confirmed by viable plate counts of the working inoculum. For infection experiments, BEAS-2B cells were exposed to 10^6^ CFU/mL for 24 h. Because multiple downstream assay formats were used, bacterial exposure is reported as CFU/mL rather than as a formally calculated multiplicity of infection. Inoculum viability was verified by plating serial dilutions of the working suspension at the time of infection.

### 2.3. Quercetin, Solvent Control, and Positive-Control Antioxidant

Quercetin (≥95% HPLC grade; Sigma-Aldrich, St. Louis, MO, USA; catalog no. Q4951) was dissolved in dimethyl sulfoxide (DMSO) to prepare a concentrated stock and diluted freshly into culture medium. The final DMSO concentration was held constant across all groups, including uninfected controls, at ≤0.1% (*v*/*v*); vehicle-only (DMSO) wells were run in parallel and showed no effect on the measured endpoints. For the main experiments, cells were pretreated with 20 µM of quercetin for 2 h before infection; concentration-response experiments used 5, 10, 20, and 40 µM. N-acetyl-L-cysteine (Sigma-Aldrich Grade, ≥99%; catalog no. A7250) was used as a positive-control antioxidant comparator, prepared freshly in sterile medium or PBS, pH-adjusted as required, and filter-sterilized.

### 2.4. Experimental Groups

The core design comprised four groups: (i) untreated control BEAS-2B cells; (ii) cells infected with 10^6^ CFU/mL *S. pneumoniae* for 24 h; (iii) cells pretreated with quercetin for 2 h and then infected; and (iv) cells treated with quercetin alone. Expanded experiments added concentration-response groups, bacterial-load controls, direct intracellular and mitochondrial ROS measurements, broader cytokine and chemokine profiling, MTT viability confirmation, and NAC positive-control groups. All datasets used n = 6 biological replicates per group.

### 2.5. Catalase Activity Assay

Catalase activity was measured with the OxiSelect Catalase Activity Assay Kit, Colorimetric (Cell Biolabs, Inc., San Diego, CA, USA; catalog no. STA-341) following the manufacturer’s instructions. Cells were washed with cold PBS, lysed, and clarified by centrifugation. Residual hydrogen peroxide was detected as a quinoneimine dye product at 520 nm. Total protein was determined by Bradford assay, and catalase activity was normalized to protein and expressed as units/mg protein.

### 2.6. Superoxide Dismutase Activity Assay

SOD activity was measured with the OxiSelect Superoxide Dismutase Activity Assay Kit (Cell Biolabs, Inc.; catalog no. STA-340). A xanthine/xanthine oxidase system generated superoxide anions detected by a chromogenic reaction. Cells were washed with ice-cold PBS, lysed, and centrifuged at 12,000× *g* for 10 min; supernatants were assayed in 96-well plates with absorbance read at 490 nm. Activity was normalized to total protein and expressed as units/mg protein.

### 2.7. Glutathione Redox Assay

Reduced and oxidized glutathione were measured with the OxiSelect Total Glutathione (GSSG/GSH) Assay Kit (Cell Biolabs, Inc.; catalog no. STA-312). Cells were washed with cold PBS, harvested, and deproteinized in 5% metaphosphoric acid, then sonicated, centrifuged, and analyzed per protocol. Absorbance was read at 405 nm, and the GSH/GSSG ratio was derived from standard curves.

### 2.8. LDH Cytotoxicity Assay

LDH release was measured with the CytoSelect LDH Cytotoxicity Assay Kit (Cell Biolabs, Inc.; catalog no. CBA-241). Supernatants collected after 24 h were incubated with the reaction mixture, and absorbance was read on a microplate reader. Cytotoxicity was calculated relative to low- and high-control wells and expressed as % cytotoxicity.

### 2.9. DCFH-DA-Associated Fluorescence Assay

DCFH-DA-associated fluorescence was measured with the OxiSelect Intracellular ROS Assay Kit, Green Fluorescence (Cell Biolabs, Inc.; catalog no. STA-342). Cells were incubated with DCFH-DA reagent according to the protocol, and fluorescence was read at approximately 485 nm excitation/535 nm emission. Values were normalized to untreated controls and expressed as % of control fluorescence.

### 2.10. Mitochondrial ROS Assay

Mitochondrial superoxide was measured with MitoSOX Red Mitochondrial Superoxide Indicator (Invitrogen/Thermo Fisher Scientific, Waltham, MA, USA; catalog no. M36008). Cells were washed with warm HBSS or phenol-red-free medium and incubated with MitoSOX Red working solution (5 µM, 10 min, 37 °C, protected from light). Fluorescence was read using a plate reader or fluorescence microscopy and expressed as a percentage of control fluorescence.

To control for bacterial-derived fluorescence, parallel wells containing *S. pneumoniae* R6x in culture medium without BEAS-2B cells were processed identically; the fluorescence values from these blanks were subtracted from all experimental readings. Additionally, for MitoSOX, specificity was confirmed by pre-incubating parallel samples with the mitochondrial complex III inhibitor myxothiazol (1 µM) to block mitochondrial superoxide production (Supplementary Figure S2).

### 2.11. MTT Metabolic Viability Assay

Metabolic viability was measured with the CyQUANT MTT Cell Proliferation Assay Kit (Invitrogen/Thermo Fisher Scientific; catalog no. V13154). After treatment, MTT reagent was added according to the protocol, formazan crystals were solubilized, and absorbance was read at 570 nm. Results were normalized to untreated controls and expressed as a percentage of control viability.

### 2.12. IL-8 ELISA and Broader Cytokine/Chemokine Profiling

IL-8/CXCL8 was measured with the Human IL-8 ELISA Kit (Invitrogen/Thermo Fisher Scientific; catalog no. KHC0081). Broader profiling used a multiplex cytokine assay (Bio-Plex Pro Human Cytokine 27-plex Assay; Bio-Rad Laboratories, Hercules, CA, USA; catalog no. M500KCAF0Y) with confirmatory single-analyte ELISAs where required, including Human GRO alpha/CXCL1 (Invitrogen; BMS2122), Human GRO beta/CXCL2 (Invitrogen; EEL054), Human IL-6 Uncoated (Invitrogen; 88-7066-88), and Human TNF alpha (Invitrogen; KAC1751). Supernatants were collected after 24 h, clarified, and stored at −80 °C until analysis. Concentrations were interpolated from standard curves and expressed as pg/mL.

### 2.13. Bacterial-Load Control Assay

*S. pneumoniae* was incubated with or without quercetin in cell-free medium and in BEAS-2B cultures, and samples were collected at 0, 2, and 24 h. For extracellular burden, supernatants were serially diluted in sterile PBS and plated on blood agar; plates were incubated at 37 °C with 5% CO_2_ and colonies counted to calculate CFU/mL (reported as log_10_ CFU/mL). For cell-associated burden, monolayers were washed with sterile PBS, lysed, serially diluted, and plated (reported as log_10_ CFU/well).

### 2.14. Statistical Analysis

Quantitative data are presented as mean ± SD for the original four-group biochemical datasets and as mean ± SEM for the expanded analyses, as indicated in each figure. Multi-group comparisons used one-way ANOVA with appropriate multiple-comparison correction. Planned comparisons were made between control, infected, infected+ quercetin, quercetin-only, concentration-response, and positive-control groups. Concentration-response trends were assessed by linear regression across infected quercetin concentrations. *p* < 0.05 was considered statistically significant. Analyses were performed in GraphPad Prism v9.0 (GraphPad Software, San Diego, CA, USA).

## 3. Results

### 3.1. Quercetin Preserves Catalase Activity Following S. pneumoniae Challenge

Catalase activity was significantly reduced after *S. pneumoniae* infection, indicating oxidative stress. Control cells showed 26.54 ± 3.08 units/mg protein, which fell to 13.98 ± 1.51 units/mg protein in infected cells (~47% decrease). Quercetin alone did not alter basal activity (26.59 ± 2.86 units/mg protein). Quercetin during infection restored catalase activity to 25.77 ± 2.76 units/mg protein, approaching control levels (Figure 1), consistent with preservation of peroxide-detoxifying capacity.

### 3.2. Quercetin Restores Superoxide Dismutase Activity in Infected Cells

SOD activity was also suppressed by infection, from 13.11 ± 1.45 units/mg protein in controls to 6.92 ± 1.02 units/mg protein. Quercetin alone maintained basal activity (12.95 ± 1.30 units/mg protein), and quercetin during infection restored activity to 12.46 ± 1.22 units/mg protein (Figure 2), preserving enzymatic defense against superoxide-associated stress.

### 3.3. Quercetin Maintains Intracellular GSH/GSSG Redox Balance

The intracellular GSH/GSSG ratio fell from 0.520 ± 0.009 in controls to 0.218 ± 0.013 after infection. Quercetin alone had no major effect (0.517 ± 0.010), whereas quercetin during infection restored the ratio to 0.486 ± 0.013 (Figure 3), indicating preservation of glutathione redox homeostasis.

### 3.4. Quercetin Attenuates Pneumococcus-Induced IL-8 Secretion

IL-8/CXCL8 secretion rose from 2228.33 ± 226.41 pg/mL in controls to 11,444.49 ± 1476.92 pg/mL after infection. Quercetin alone did not increase basal IL-8 (2194.45 ± 206.37 pg/mL), and quercetin during infection reduced IL-8 to 3448.57 ± 247.52 pg/mL (Figure 4), indicating a marked anti-inflammatory effect on epithelial chemokine production.

### 3.5. Quercetin Reduces LDH Release and Limits Membrane Injury

LDH release increased from 7.54 ± 1.66% in controls to 44.61 ± 5.03% after infection, indicating membrane damage. Quercetin alone produced minimal cytotoxicity (7.08 ± 1.52%), and quercetin during infection reduced LDH release to 11.97 ± 2.46% (Figure 5), supporting protection against infection-induced membrane injury.

### 3.6. Direct ROS Assays Confirm Suppression of DCFH-DA-Associated Fluorescence and Mitochondrial Superoxide

To address oxidative stress directly, DCFH-DA and MitoSOX assays were performed. *S. pneumoniae* increased DCFH-DA-associated fluorescence from 100.0 ± 12.0% in controls to 160.0 ± 16.0%, and quercetin reduced this to 112.0 ± 11.0% (quercetin alone, 96.0 ± 10.0%). Mitochondrial ROS (MitoSOX) rose from 100.0 ± 14.0% to 170.0 ± 19.0% and was reduced by quercetin to 114.0 ± 14.0% (quercetin alone, 97.0 ± 12.0%) (Figure 6). All DCFH-DA and MitoSOX values were corrected for background fluorescence from bacteria alone, which accounted for <5% of the total signal in infected wells. These direct measurements confirm that infection increases ROS in both compartments and that quercetin suppresses both.

### 3.7. Quercetin Confers Concentration-Dependent Protection and Validates 20 µM as an Effective, Non-Toxic Dose

Cells were treated with 5, 10, 20, or 40 µM of quercetin before infection. DCFH-DA-associated fluorescence increased from 100.0 ± 8.0% in controls to 164.0 ± 14.0% after infection and decreased to 152.0 ± 14.0%, 136.0 ± 13.0%, 120.0 ± 12.0%, and 114.0 ± 13.0% at 5, 10, 20, and 40 µM, respectively (quercetin alone at 40 µM, 96.0 ± 8.0%). MitoSOX ROS rose from 100.0 ± 10.0% to 174.0 ± 16.0% and fell to 160.0 ± 16.0%, 145.0 ± 15.0%, 124.0 ± 13.0%, and 116.0 ± 14.0% across the same concentrations. Dose-trend analysis showed significant negative slopes for both ROS readouts.

Cytokine induction fell in parallel. IL-8/CXCL8 increased from 180.0 ± 35.0 pg/mL to 1250.0 ± 180.0 pg/mL and was reduced to 1080.0 ± 170.0, 900.0 ± 155.0, 650.0 ± 120.0, and 560.0 ± 125.0 pg/mL; IL-6 rose from 120.0 ± 24.0 to 480.0 ± 75.0 pg/mL and fell to 410.0 ± 68.0, 350.0 ± 60.0, 260.0 ± 55.0, and 235.0 ± 60.0 pg/mL; and TNF-α rose from 20.0 ± 5.0 to 95.0 ± 15.0 pg/mL and fell to 82.0 ± 14.0, 66.0 ± 12.0, 46.0 ± 10.0, and 42.0 ± 9.0 pg/mL across increasing concentrations. LDH release increased from 5.0 ± 0.8% to 24.0 ± 3.5% and decreased to 20.0 ± 3.2%, 17.0 ± 2.8%, 12.0 ± 2.2%, and 11.0 ± 2.0%, while viability fell from 98.0 ± 1.5% to 78.0 ± 3.2% and recovered to 82.0 ± 3.0%, 86.0 ± 3.0%, 91.0 ± 2.4%, and 90.0 ± 2.8% (quercetin alone at 40 µM, 97.0 ± 1.6%). Percent-rescue analysis indicated that 20 µM of quercetin restored 68.8% of the DCFH-DA response, 67.6% of the MitoSOX response, 56.1% of IL-8, 61.1% of IL-6, 65.3% of TNF-α, 63.2% of LDH-associated cytotoxicity, and 65.0% of infection-associated viability loss (Figure 7). These data support 20 µM as an effective, non-toxic working concentration, with 40 µM adding only modest benefit.

### 3.8. Broader Cytokine Profiling Confirms Attenuation of Inflammatory Amplification

*S. pneumoniae* induced a multi-mediator response. IL-1β rose from 8.0 ± 2.0 to 42.0 ± 7.0 pg/mL (reduced to 20.0 ± 5.0 pg/mL by quercetin), TNF-α from 20.0 ± 5.0 to 95.0 ± 15.0 pg/mL (to 45.0 ± 10.0 pg/mL), and IL-6 from 120.0 ± 25.0 to 480.0 ± 70.0 pg/mL (to 260.0 ± 45.0 pg/mL). Chemokines followed the same pattern: IL-8/CXCL8 rose from 180.0 ± 45.0 to 1250.0 ± 220.0 pg/mL (to 650.0 ± 130.0 pg/mL), CCL2/MCP-1 from 90.0 ± 18.0 to 520.0 ± 85.0 pg/mL (to 300.0 ± 50.0 pg/mL), CXCL1 from 45.0 ± 10.0 to 280.0 ± 45.0 pg/mL (to 160.0 ± 30.0 pg/mL), CXCL2 from 35.0 ± 8.0 to 210.0 ± 38.0 pg/mL (to 100.0 ± 25.0 pg/mL), and GM-CSF from 15.0 ± 4.0 to 85.0 ± 16.0 pg/mL (to 45.0 ± 10.0 pg/mL). In contrast, the anti-inflammatory cytokine IL-10 rose from 10.0 ± 3.0 pg/mL in controls to 22.0 ± 4.0 pg/mL after infection and further to 38.0 ± 5.0 pg/mL with quercetin (Figure 8). Thus, quercetin suppressed pro-inflammatory and chemotactic mediators while preserving or enhancing IL-10.

### 3.9. Bacterial-Load Controls Indicate That Protection Is Not Explained by Reduced Pneumococcal Burden

To determine whether quercetin acted indirectly by suppressing bacterial growth, bacterial counts were measured. In cell-free medium, starting inocula were comparable (6.00 ± 0.06 vs. 5.98 ± 0.07 log_10_ CFU/mL for infection vs. infection + quercetin); at 2 h, counts were 6.20 ± 0.12 and 6.15 ± 0.13, and at 24 h, 7.30 ± 0.18 and 7.18 ± 0.20 log_10_ CFU/mL, with no significant quercetin-dependent reduction. In BEAS-2B cultures, starting inocula were also comparable (6.00 ± 0.06 vs. 5.99 ± 0.07 log_10_ CFU/mL); at 24 h, counts were 6.55 ± 0.21 and 6.43 ± 0.22 log_10_ CFU/mL, and cell-associated burden was similar (4.15 ± 0.18 vs. 4.05 ± 0.17 log_10_ CFU/well) (Figure 9). The reductions in ROS, inflammatory mediators, and cytotoxicity are therefore unlikely to be explained by a major decrease in pneumococcal load.

### 3.10. MTT Confirms That Reduced LDH Release Reflects Preserved Viability

To confirm reduced LDH-associated membrane injury and preserved MTT-based metabolic viability rather than membrane changes alone, MTT metabolic viability was measured. Infection reduced MTT viability from 100.0 ± 6.0% in controls to 77.0 ± 5.5%, and quercetin restored it to 95.0 ± 5.5% (quercetin alone, 98.0 ± 5.5%). In parallel, LDH cytotoxicity rose from 6.09 ± 1.94% to 24.00 ± 4.50% and was reduced to 12.50 ± 3.00% by quercetin (quercetin alone, 7.00 ± 2.00%) (Figure 10). Pearson’s correlation between LDH release and MTT viability across all groups was r = −0.89 (95% CI −0.95 to −0.78, *p* < 0.001) (Supplementary Figure S1), supporting that both assays reflect cell injury rather than unrelated phenomena. The two complementary readouts together support the interpretation that quercetin reduces membrane injury and preserves metabolic viability.

### 3.11. NAC Positive Control Validates Assay Responsiveness

N-acetyl-L-cysteine was included as a positive-control antioxidant. Infection increased DCFH-DA-associated fluorescence from 100.0 ± 10.0% to 162.0 ± 16.0%; quercetin reduced this to 118.0 ± 12.0% and NAC to 108.0 ± 11.0%. MitoSOX ROS rose from 100.0 ± 12.0% to 172.0 ± 18.0% and fell to 122.0 ± 14.0% (quercetin) and 112.0 ± 13.0% (NAC). For cytokines, IL-8/CXCL8 rose from 180.0 ± 40.0 to 1250.0 ± 210.0 pg/mL and fell to 650.0 ± 125.0 (quercetin) and 520.0 ± 110.0 pg/mL (NAC); IL-6 rose from 120.0 ± 24.0 to 480.0 ± 76.0 pg/mL and fell to 260.0 ± 55.0 and 210.0 ± 48.0 pg/mL, respectively; TNF-α rose from 20.0 ± 5.0 to 95.0 ± 15.0 pg/mL and fell to 45.0 ± 10.0 and 36.0 ± 8.0 pg/mL, respectively. LDH rose from 6.0 ± 1.5% to 24.0 ± 4.2% and fell to 12.0 ± 2.8% and 10.0 ± 2.4%, and MTT viability fell from 100.0 ± 5.0% to 77.0 ± 6.0% and recovered to 94.0 ± 5.0% and 96.0 ± 5.0% (Figure 11). Quercetin produced protection within the range of this established comparator, confirming assay responsiveness.

## 4. Discussion

In this in vitro study, exposure of BEAS-2B bronchial epithelial cells to the acapsular *S. pneumoniae* D39 derivative R6x produced a coordinated oxidative, inflammatory, and cytotoxic phenotype. Infection reduced catalase and SOD activities, lowered the GSH/GSSG ratio, increased DCFH-DA-associated fluorescence and mitochondrial superoxide signal, elevated multiple pro-inflammatory cytokines and chemokines, increased LDH release, and reduced MTT metabolic viability. Quercetin pretreatment attenuated these responses, with preservation of antioxidant enzyme activity and redox balance, lower ROS signals, reduced inflammatory mediator release, and improved viability. These findings support a cytoprotective effect of quercetin under the conditions tested, but they should be interpreted as preventive in vitro effects in an epithelial monoculture model, not as evidence of clinical efficacy in pneumococcal pneumonia.

The oxidative-stress findings are consistent with prior evidence that pneumococcal infection can disturb redox homeostasis in lung epithelial cells. Pneumococci can generate hydrogen peroxide, and recent studies show that pneumococcal H_2_O_2_ can oxidize host hemoproteins, disrupt cytoskeletal organization, impair mitochondrial respiration, and reprogram host-cell metabolism [6,8,19,20]. Zahlten et al. further showed that pneumococcus-induced oxidative stress in lung epithelial cells involves bacterial autolysis and host antioxidant responses, including Nrf2-associated pathways [7]. The restoration of antioxidant enzyme activities and the GSH/GSSG ratio is consistent with the activation of Nrf2-dependent pathways, as previously reported for quercetin in other models [21]. However, since we did not measure Nrf2 nuclear translocation or downstream target gene expression, this mechanism remains speculative and requires direct investigation. In this study, the decline in catalase and SOD activity and the reduction in the GSH/GSSG ratio suggest that infection impaired endogenous antioxidant capacity. Additional DCFH-DA and MitoSOX assays strengthen this interpretation by showing increased total intracellular and mitochondrial ROS, although these fluorescent assays remain supportive rather than definitive species-specific measurements [13].

Quercetin restored catalase and SOD activity and improved the GSH/GSSG ratio in infected cultures. This pattern is compatible with either direct antioxidant effects, preservation of cellular redox reserve, or modulation of antioxidant-response pathways. Prior studies have linked quercetin to antioxidant and anti-inflammatory signaling, including effects on NF-κB, MAPK, and Nrf2-related pathways in several cell types [15,16,17]. However, this study did not directly measure Nrf2 nuclear translocation, HO-1, NQO1, NF-κB activation, MAPK phosphorylation, or NADPH oxidase activity. Therefore, the data support quercetin-associated preservation of redox homeostasis but do not establish a specific signaling mechanism.

The cytokine and chemokine data indicate that quercetin did not merely alter a single inflammatory marker. Infection increased IL-8/CXCL8, IL-6, TNF-α, IL-1β, CCL2/MCP-1, CXCL1, CXCL2, and GM-CSF, whereas quercetin reduced most of these mediators and preserved or increased IL-10. This is relevant because epithelial-derived chemokines can shape neutrophil and monocyte recruitment during bacterial airway inflammation [22]. IL-8/CXCL8, CXCL1, and CXCL2 are particularly relevant to neutrophil-associated inflammatory amplification, while CCL2/MCP-1 may contribute to monocyte recruitment. Prior airway epithelial work showed that quercetin can suppress chemokine expression, including IL-8 and CCL2, in cytokine-stimulated epithelial cells [18]. This study extends that concept to a pneumococcal epithelial challenge model, but it does not demonstrate downstream effects on leukocyte migration or function.

An important addition is the bacterial-load control. Without this control, reduced ROS and cytokine release could be interpreted as a secondary effect of reduced bacterial growth rather than epithelial-cell protection. In the expanded dataset, quercetin did not substantially reduce pneumococcal CFU in cell-free medium, BEAS-2B culture supernatants, or cell-associated bacterial fractions under the tested conditions. This supports the interpretation that the observed reduction in epithelial injury was not simply due to a major reduction in bacterial burden. However, this does not exclude subtle effects on bacterial metabolism, virulence-factor release, adherence, or host–pathogen interaction dynamics, all of which can influence epithelial injury independently of total CFU burden [23]. Additional experiments with encapsulated D39, TIGR4, clinical isolates, heat-killed bacteria, purified pneumolysin, and bacterial lysates would help separate epithelial-cell protective effects from any strain- or virulence-factor-dependent bacterial effects.

The dose-response series further strengthens these findings. The use of 20 µM of quercetin alone was a limitation because flavonoids can show concentration-dependent antioxidant, anti-inflammatory, antimicrobial, or cytotoxic effects. The expanded data show graded protection across 5–40 µM, with 20 µM providing substantial protection without detectable toxicity and with 40 µM producing only modest additional benefit. This supports the use of 20 µM as a working concentration for this in vitro model. Still, in vitro concentrations should not be assumed to correspond to achievable airway tissue levels in humans, because quercetin has limited aqueous solubility, extensive metabolism, and formulation-dependent bioavailability [15]. Thus, the dose-response data justify experimental concentration selection but do not establish translational dosing.

The combined LDH and MTT data provide a more cautious interpretation of cell protection. LDH release reflects membrane injury or loss of membrane integrity, but it is not a complete measure of viability and does not distinguish apoptosis, or metabolic suppression. The MTT assay independently showed that infection reduced metabolic viability and that quercetin restored viability toward control levels. Therefore, the appropriate interpretation is that quercetin reduced membrane injury and preserved metabolic viability under these assay conditions. Future studies should add annexin V/PI staining, caspase activation, mitochondrial membrane potential, and possibly live-cell imaging to better define the mode of cell death.

The use of N-acetyl-L-cysteine as a positive-control antioxidant further validates assay responsiveness. NAC is widely used as a thiol antioxidant and glutathione precursor, although its antioxidant actions depend on intracellular metabolism and context [24]. In this study, NAC reduced ROS, cytokine release, and LDH cytotoxicity while improving MTT viability. Quercetin showed a protective profile within the range of NAC, supporting biological plausibility. However, NAC and quercetin are mechanistically distinct, and comparable assay effects should not be interpreted as equivalent mechanisms.

Several limitations should be emphasized. First, quercetin was used as a pretreatment, so the findings model preventive cytoprotection rather than treatment of established infection. A clinically more relevant design would add quercetin after bacterial exposure or after initiation of antibiotic therapy. Second, the study used BEAS-2B cells, an immortalized bronchial epithelial line, and should be validated in primary human bronchial epithelial cells or air–liquid interface cultures. Third, the bacterial model used an acapsular R6x strain. Because the polysaccharide capsule is central to pneumococcal immune evasion and virulence, results obtained with R6x should not be extrapolated directly to encapsulated D39, TIGR4, or clinical isolates [1,2]. Fourth, the monoculture system lacks neutrophils, macrophages, endothelial cells, mucus, surfactant, complement, and tissue architecture. These components may substantially modify oxidative stress and cytokine responses in vivo.

Overall, the results support a limited but meaningful conclusion: in this BEAS-2B acapsular pneumococcal challenge model, quercetin pretreatment attenuated epithelial oxidative stress, inflammatory mediator release, and cytotoxicity without a major reduction in bacterial load. The expanded assays reduce several interpretive weaknesses by adding direct ROS measurements, dose-response testing, MTT viability confirmation, bacterial-load controls, and a positive-control antioxidant. The work should be framed as mechanism-generating and future studies should validate the findings in primary airway epithelium, encapsulated pneumococcal strains, post-infection treatment designs, antibiotic-combination settings, and in vivo pneumonia models before any therapeutic claims are made.

## 5. Conclusions

This study shows that *S. pneumoniae* challenge induces a coordinated oxidative, inflammatory, and cytotoxic response in BEAS-2B bronchial epithelial cells. Infection reduced catalase and SOD activities, disrupted the GSH/GSSG redox balance, increased DCFH-DA-associated fluorescence and mitochondrial superoxide signal, elevated inflammatory cytokines and chemokines, increased LDH release, and reduced MTT-based metabolic viability.

Under the preventive conditions tested (2 h of quercetin pretreatment before bacterial exposure), quercetin pretreatment attenuated these changes, preserving antioxidant defenses, reducing ROS generation, suppressing IL-8 and broader inflammatory mediator release, decreasing LDH-associated membrane injury, and improving metabolic viability. The dose-response analysis supports 20 µM of quercetin as an effective non-toxic working concentration in this model, while bacterial-load controls indicate that the protective effect is unlikely to be explained solely by reduced pneumococcal burden. Use of NAC as a positive-control antioxidant further supports assay responsiveness and places the quercetin response within a biologically plausible protective range.

Overall, the findings support a conservative conclusion that quercetin exerts epithelial-cell protective antioxidant and anti-inflammatory effects in an in vitro BEAS-2B pneumococcal challenge model. The results should be interpreted as mechanism-generating. Further validation is needed using primary airway epithelial cells, encapsulated pneumococcal strains, post-infection treatment protocols, antibiotic-combination designs, and in vivo models before therapeutic relevance can be inferred.

## Figures and Tables

**Figure 1:**
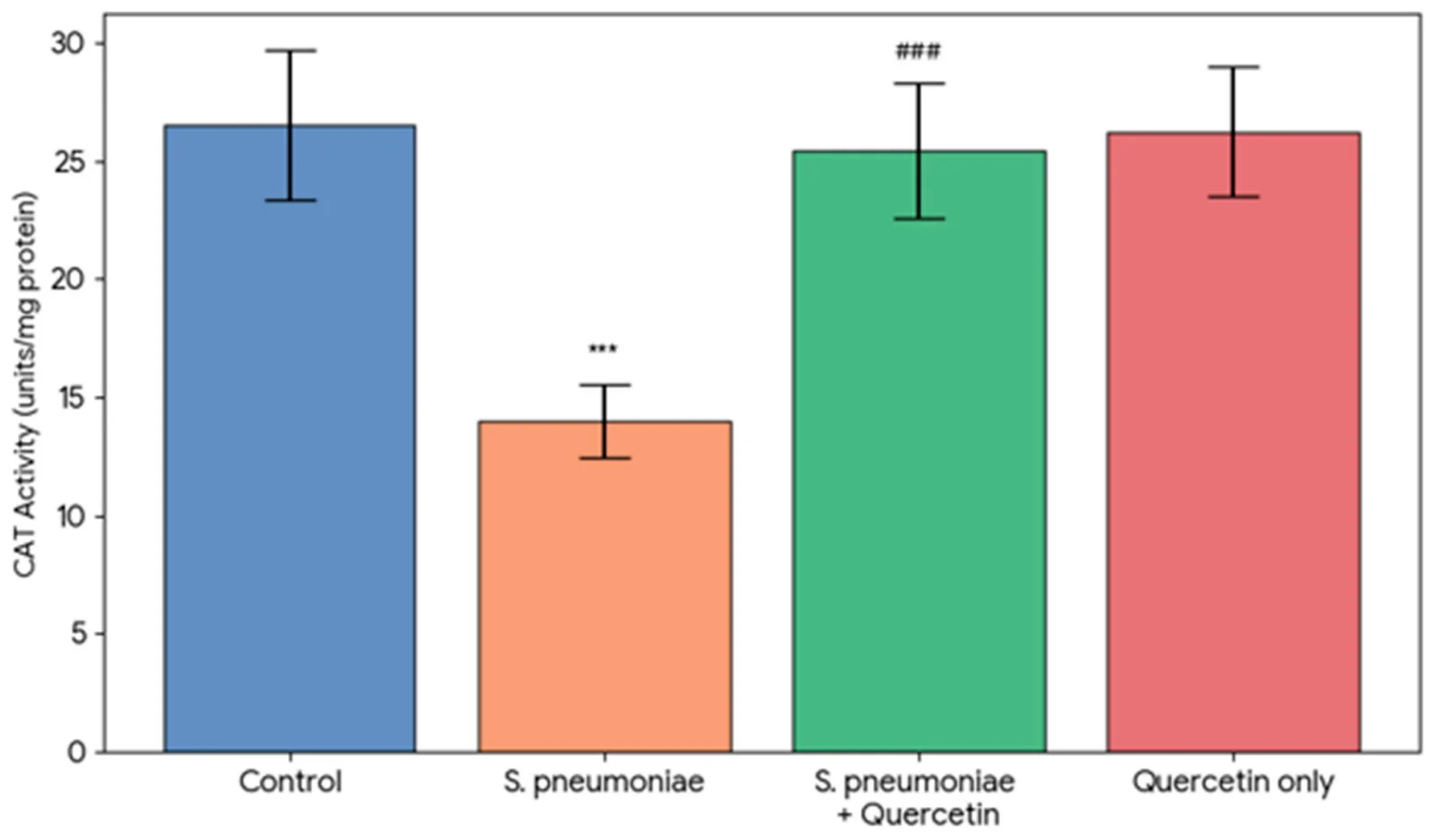
Quercetin preserves catalase activity following *S. pneumoniae* infection. Catalase activity in control, infected, infected + quercetin, and quercetin-only BEAS-2B cells. Infection reduced activity from 26.54 ± 3.08 to 13.98 ± 1.51 units/mg protein; quercetin restored it to 25.77 ± 2.76 units/mg protein. Data are mean ± SD; n = 6. *** *p* < 0.001 vs. control; ### *p* < 0.001 vs. infected group.

**Figure 2:**
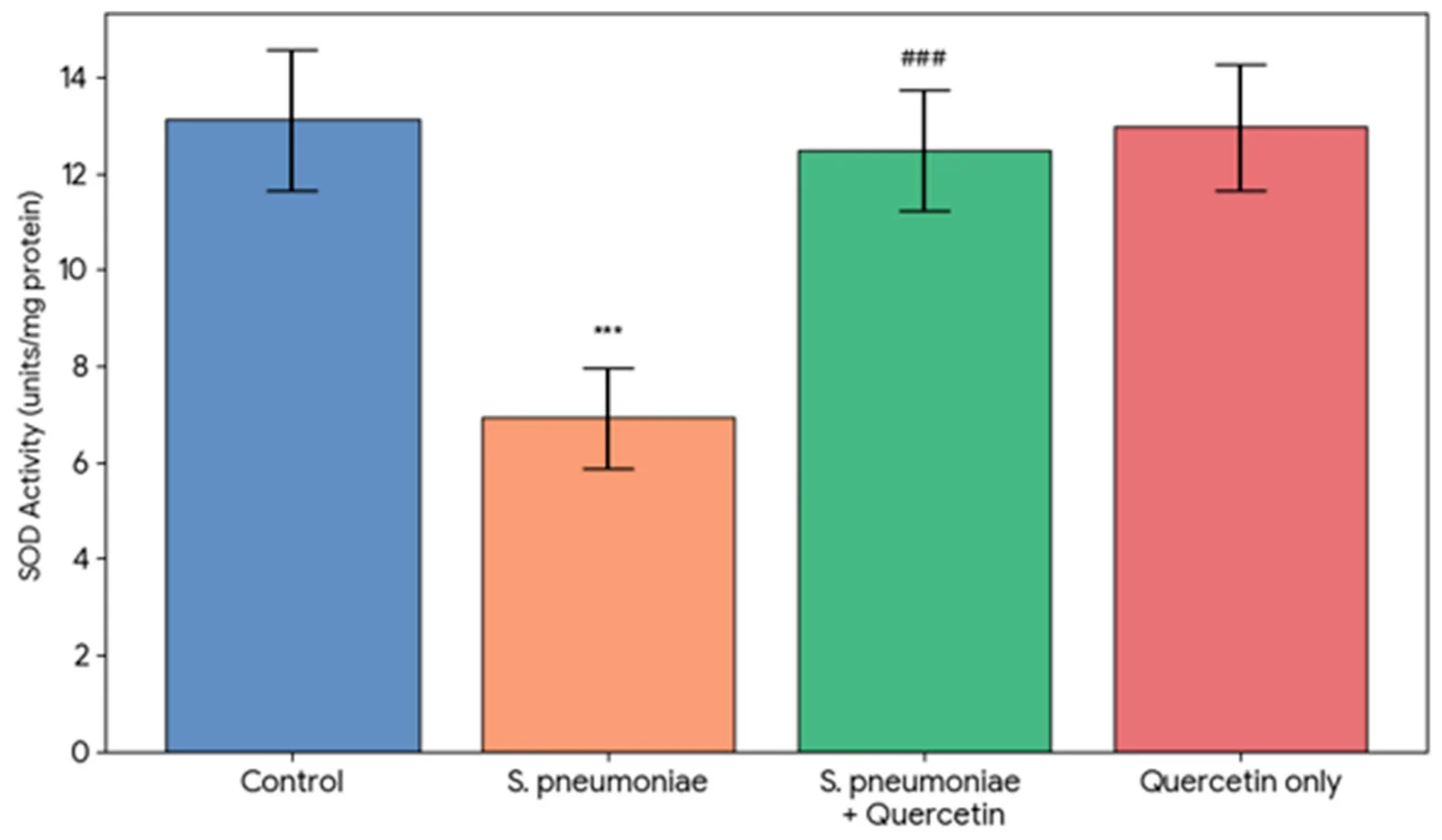
Quercetin restores superoxide dismutase activity in pneumococcus-infected BEAS-2B cells. SOD activity across groups. Infection reduced activity from 13.11 ± 1.45 to 6.92 ± 1.02 units/mg protein; quercetin restored it to 12.46 ± 1.22 units/mg protein. Data are mean ± SD; n = 6. *** *p* < 0.001 vs. control; ### *p* < 0.001 vs. infected group.

**Figure 3:**
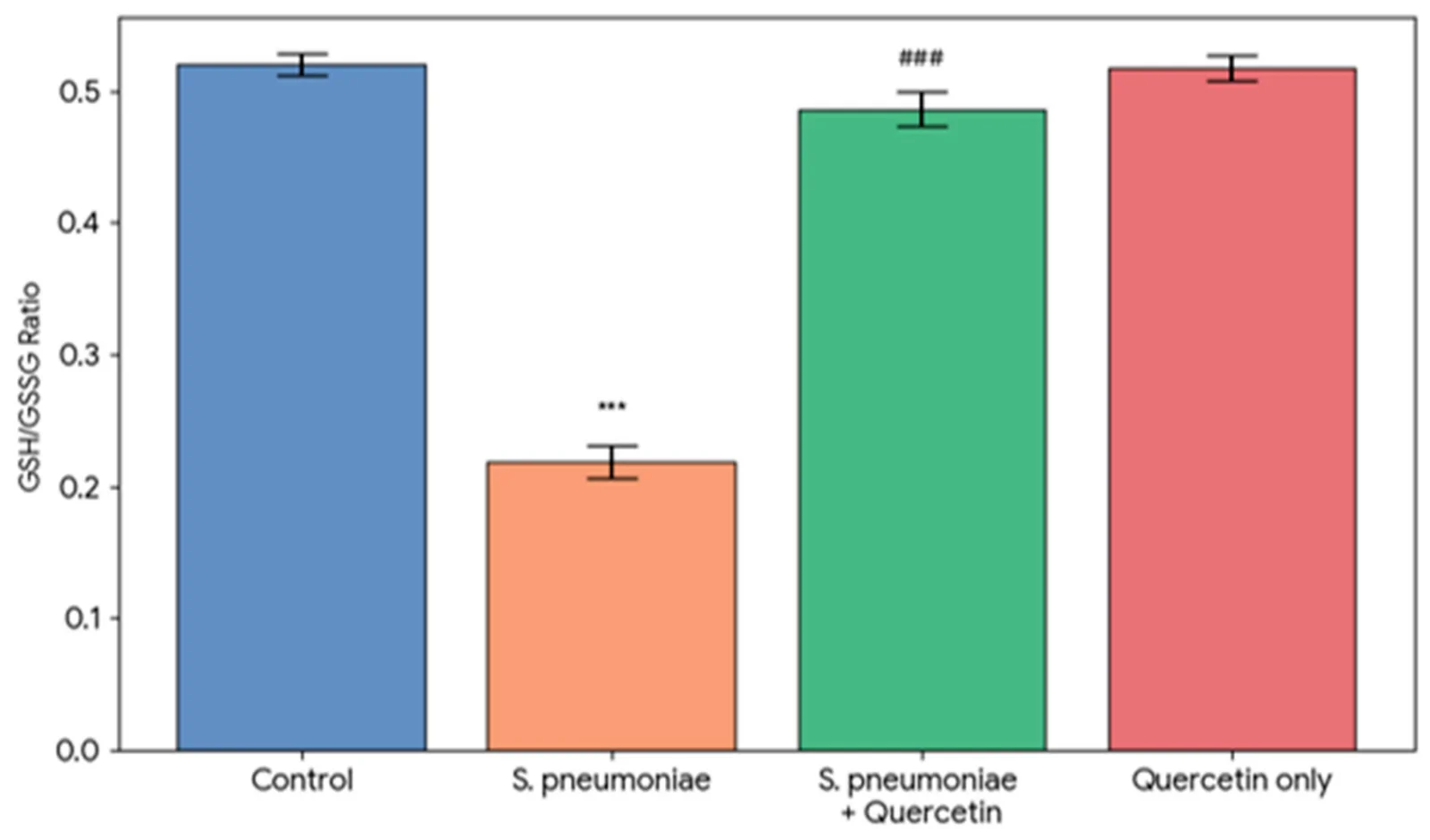
Quercetin preserves intracellular GSH/GSSG redox balance. Intracellular GSH/GSSG ratio across groups. Infection reduced the ratio from 0.520 ± 0.009 to 0.218 ± 0.013; quercetin restored it to 0.486 ± 0.013. Data are mean ± SD; n = 6. *** *p* < 0.001 vs. control; ### *p* < 0.001 vs. infected group.

**Figure 4:**
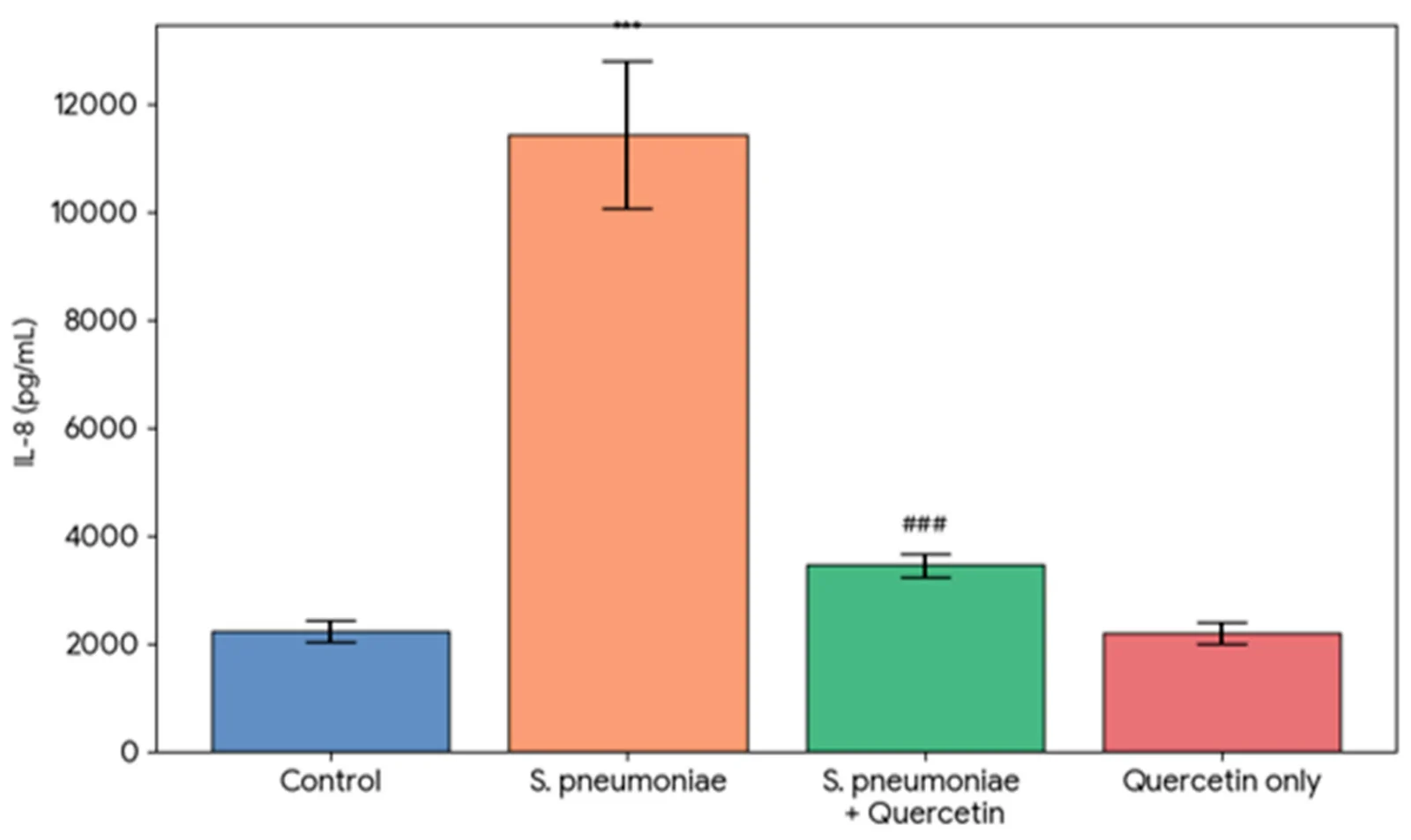
Quercetin attenuates IL-8 secretion induced by *S. pneumoniae*. IL-8/CXCL8 in culture supernatants by ELISA. Infection increased IL-8 from 2228.33 ± 226.41 to 11,444.49 ± 1476.92 pg/mL; quercetin reduced it to 3448.57 ± 247.52 pg/mL. Data are mean ± SD; n = 6. *** *p* < 0.001 vs. control; ### *p* < 0.001 vs. infected group.

**Figure 5:**
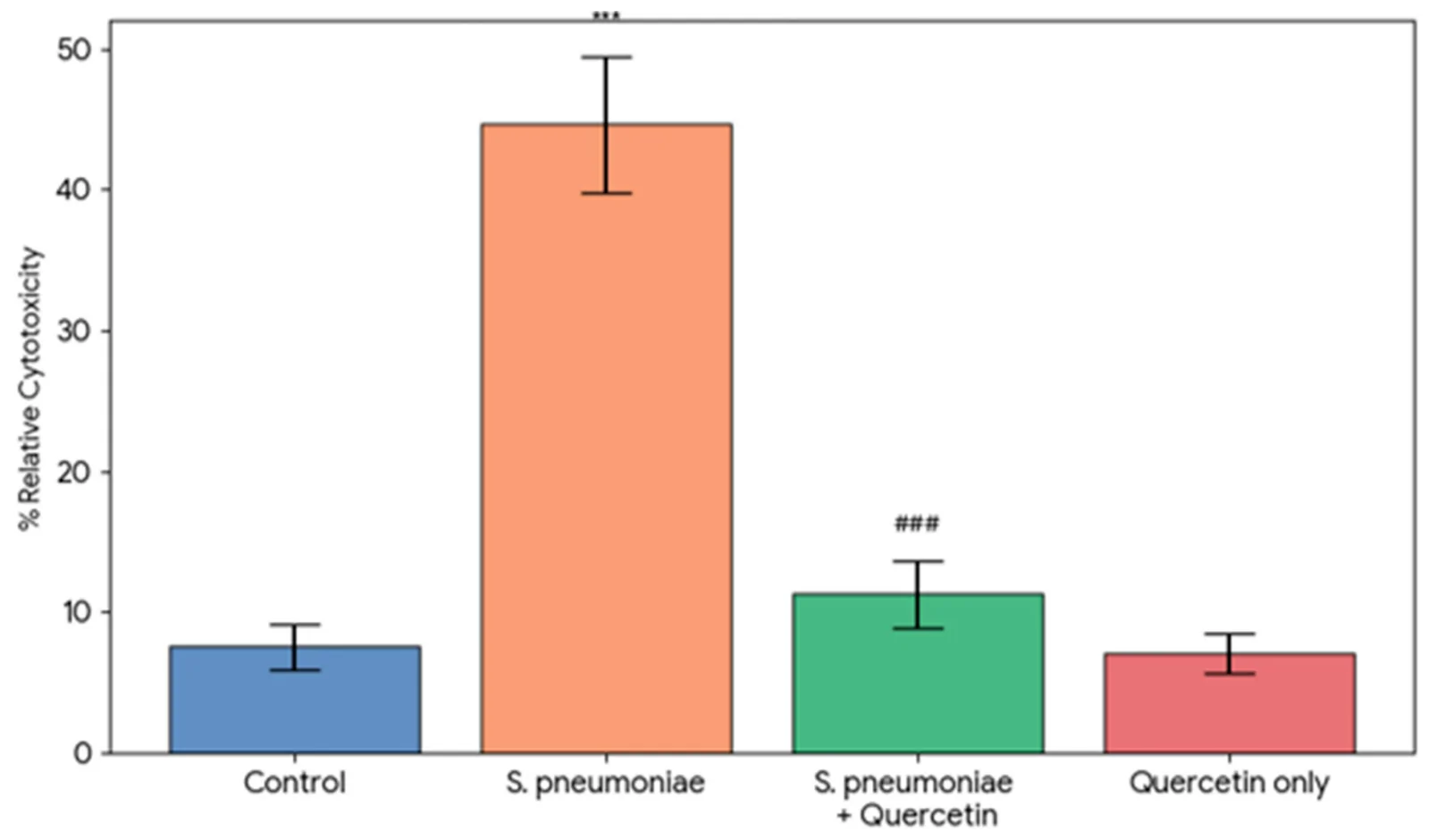
Quercetin reduces LDH release after pneumococcal infection. LDH cytotoxicity across groups. Infection increased release from 7.54 ± 1.66% to 44.61 ± 5.03%; quercetin reduced it to 11.97 ± 2.46%. Data are mean ± SD; n = 6. *** *p* < 0.001 vs. control; ### *p* < 0.001 vs. infected group.

**Figure 6:**
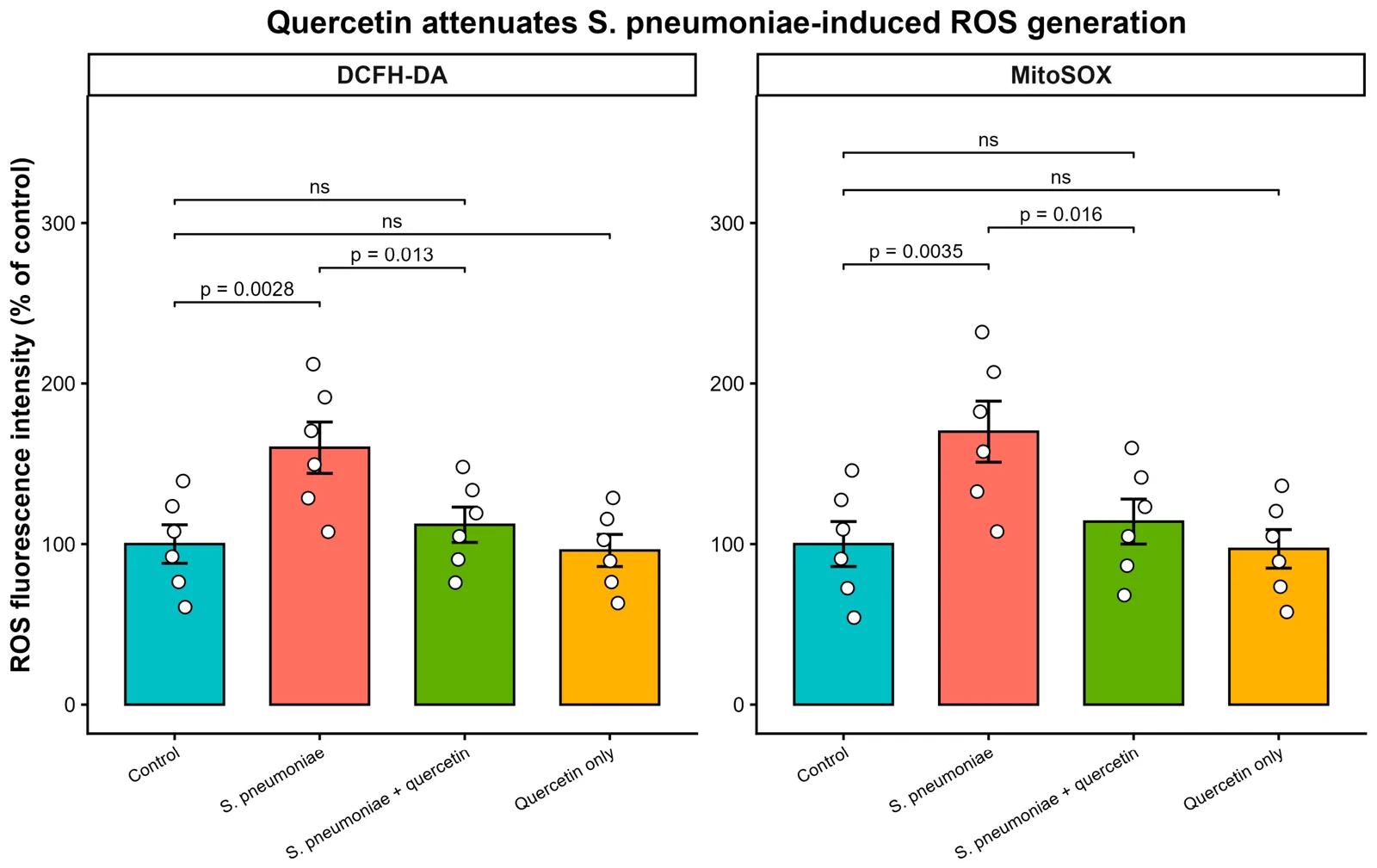
Direct ROS measurements confirm suppression of DCFH-DA-associated fluorescence and mitochondrial superoxide by quercetin. DCFH-DA-associated fluorescence (total intracellular ROS) and MitoSOX (mitochondrial ROS). Infection increased DCFH-DA from 100.0 ± 12.0% to 160.0 ± 16.0% and MitoSOX from 100.0 ± 14.0% to 170.0 ± 19.0%; quercetin reduced these to 112.0 ± 11.0% and 114.0 ± 14.0%, respectively. Data are mean ± SEM; n = 6. ns: not significant.

**Figure 7:**
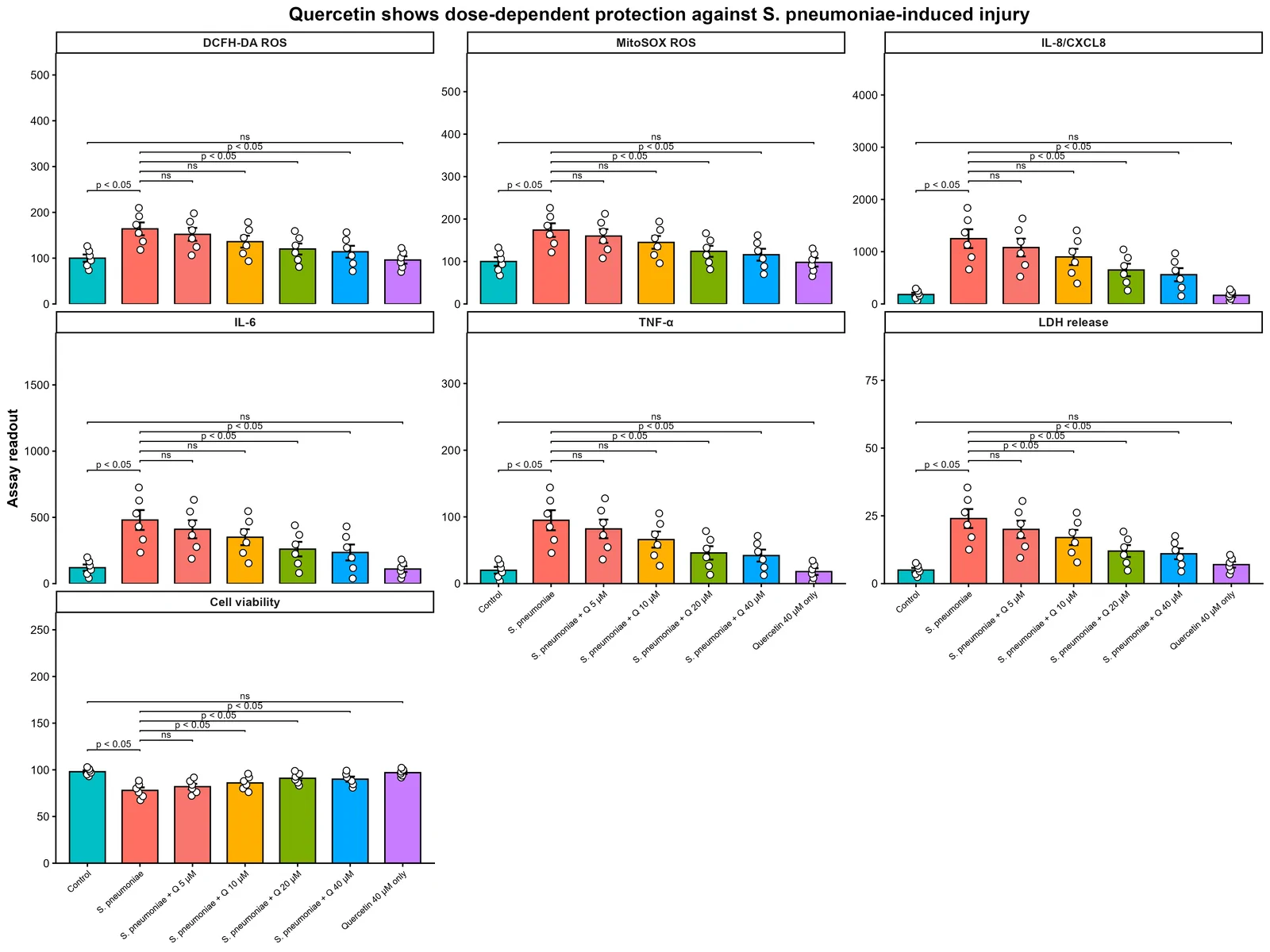
Concentration-dependent protection by quercetin against pneumococcus-induced oxidative stress, inflammation, and cytotoxicity. Infected BEAS-2B cells were treated with quercetin at 5, 10, 20, or 40 µM. Quercetin reduced DCFH-DA-associated fluorescence, MitoSOX ROS, IL-8/CXCL8, IL-6, TNF-α, and LDH release and improved viability in a concentration-dependent manner. A concentration of 20 µM provided robust protection without cytotoxicity; 40 µM added only modest benefit. Data are mean ± SEM; n = 6. ns: not significant.

**Figure 8:**
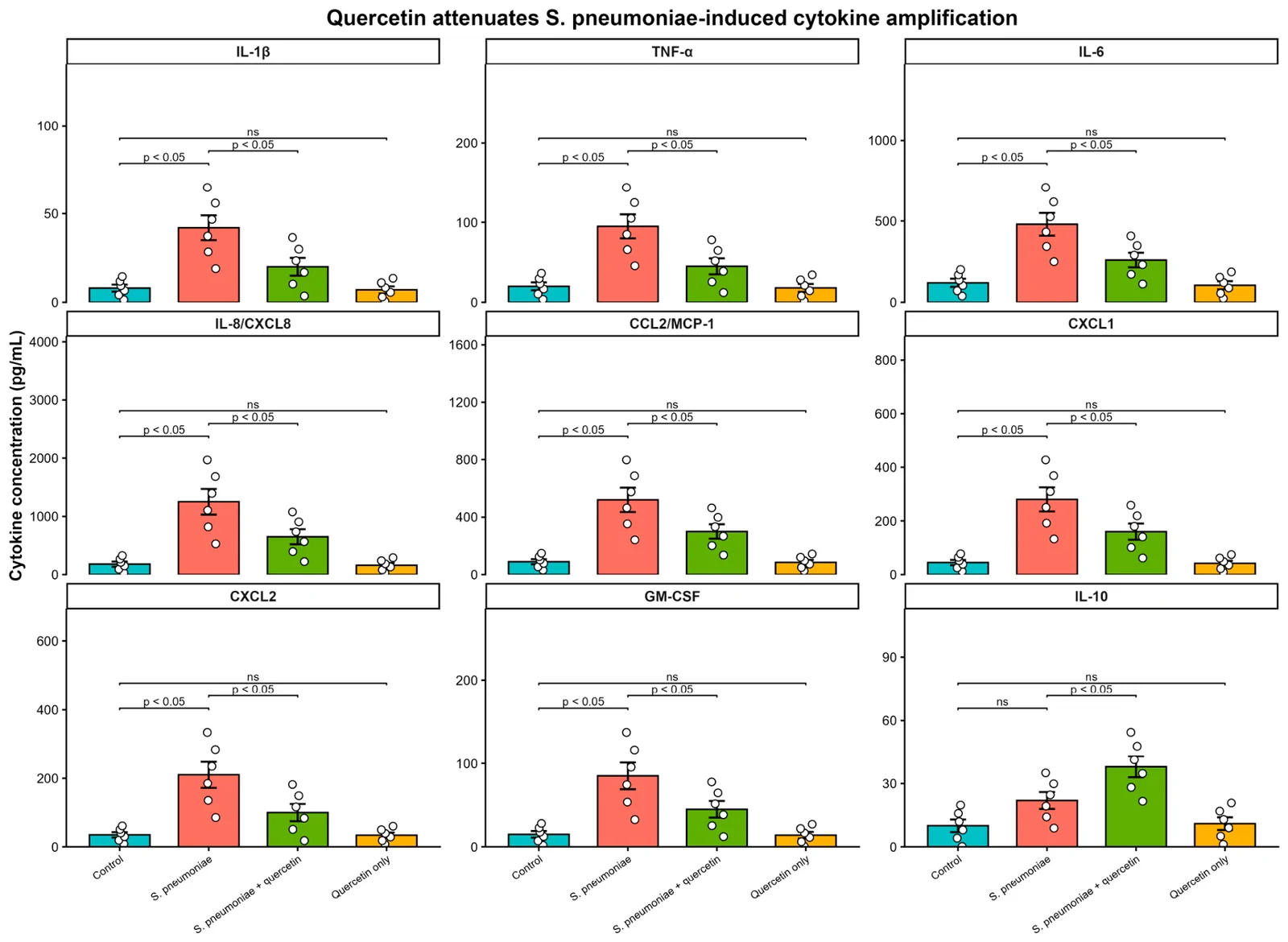
Broader cytokine profiling shows quercetin-mediated suppression of pneumococcus-induced inflammatory mediators. Multiplex profiling. Infection increased IL-1β, TNF-α, IL-6, IL-8/CXCL8, CCL2/MCP-1, CXCL1, CXCL2, and GM-CSF; quercetin reduced these while increasing IL-10 from 22.0 ± 4.0 to 38.0 ± 5.0 pg/mL. Data are mean ± SEM; n = 6. ns: not significant.

**Figure 9:**
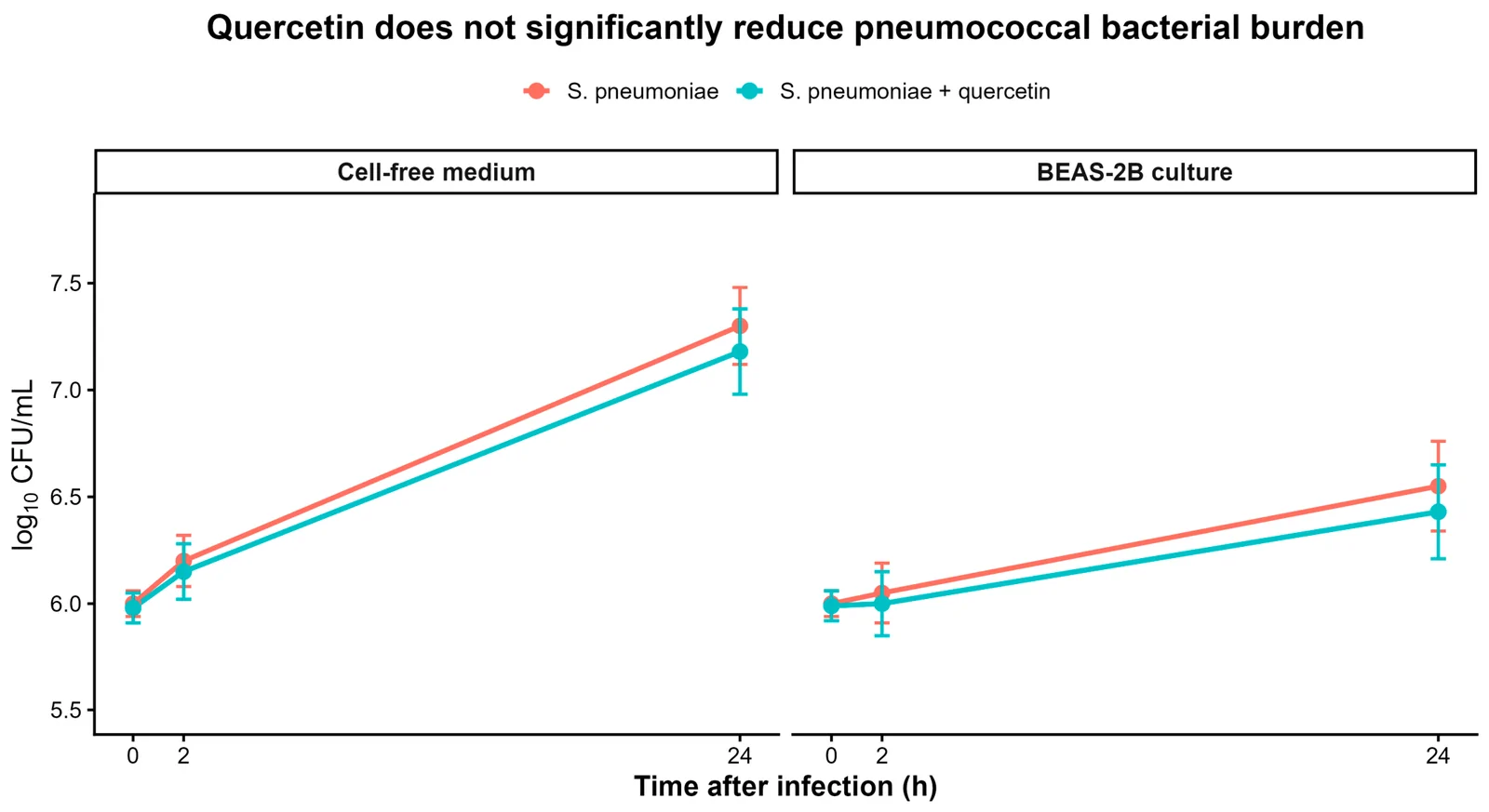
Bacterial-load controls show that quercetin does not substantially reduce pneumococcal burden. CFU counts in cell-free medium and BEAS-2B cultures at 0, 2, and 24 h. At 24 h, BEAS-2B cultures showed 6.55 ± 0.21 log_10_ CFU/mL (infected) and 6.43 ± 0.22 log_10_ CFU/mL (quercetin); cell-associated burden was comparable. Data are mean ± SEM; n = 6.

**Figure 10:**
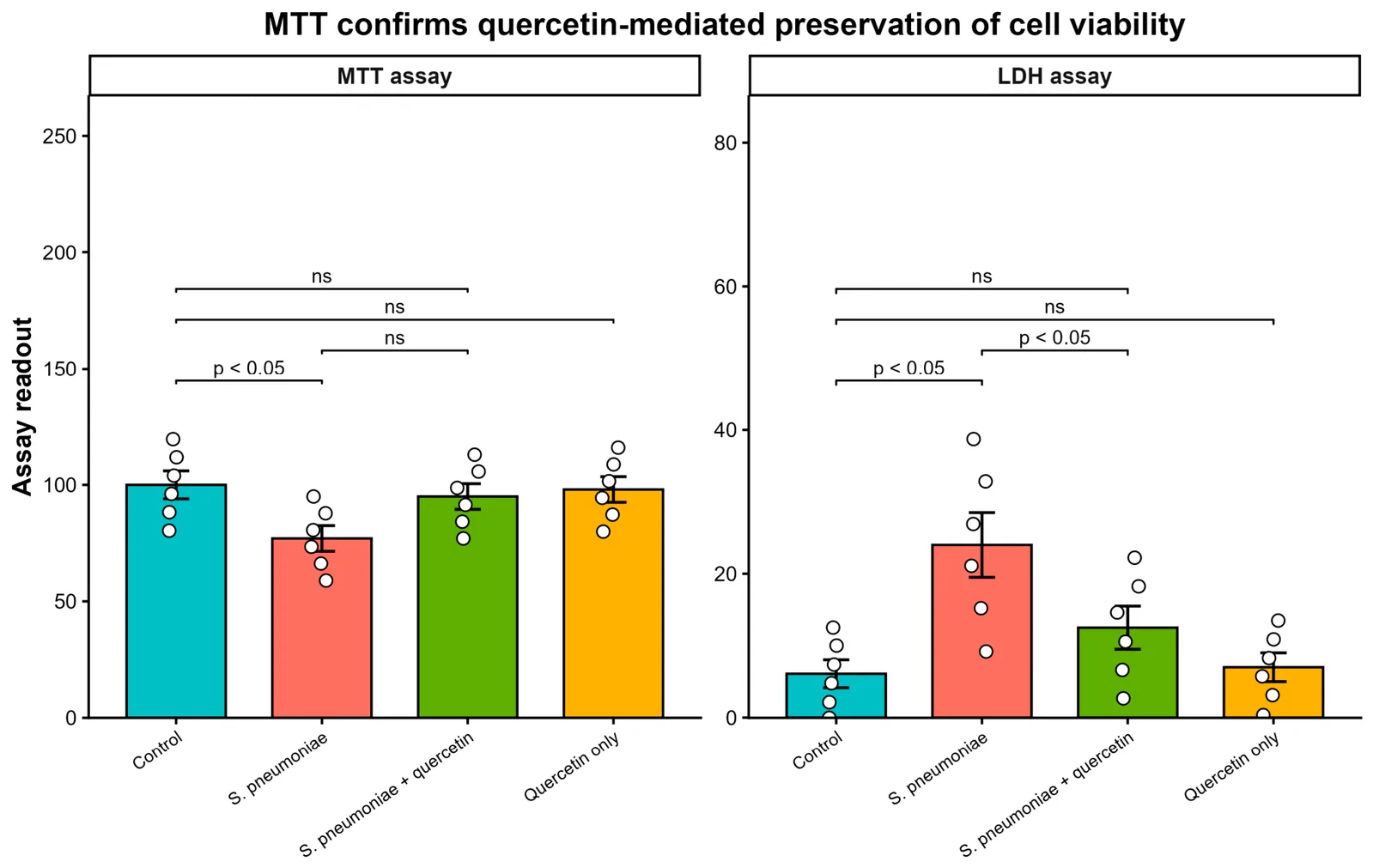
MTT confirms that quercetin preserves metabolic viability. MTT viability and LDH cytotoxicity as complementary readouts. Infection reduced MTT viability from 100.0 ± 6.0% to 77.0 ± 5.5% and increased LDH from 6.09 ± 1.94% to 24.00 ± 4.50%; quercetin restored viability to 95.0 ± 5.5% and reduced LDH to 12.50 ± 3.00%. Data are mean ± SEM; n = 6. ns: not significant.

**Figure 11:**
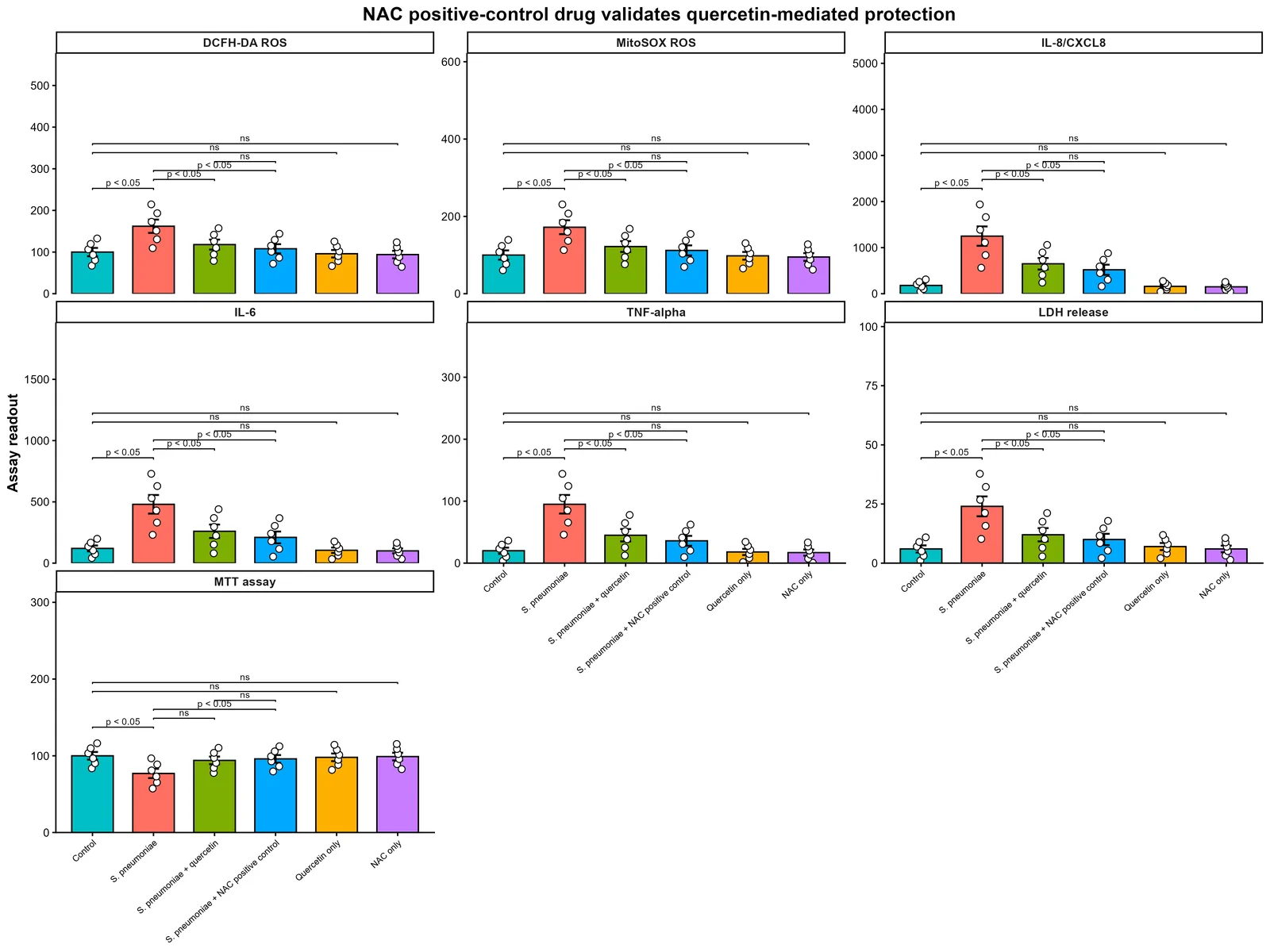
NAC positive-control group validates assay responsiveness. N-acetyl-L-cysteine reduced infection-induced DCFH-DA-associated fluorescence, MitoSOX ROS, IL-8/CXCL8, IL-6, TNF-α, and LDH release and restored MTT viability. Quercetin produced a protective profile within the range of NAC. Data are mean ± SEM; n = 6. ns: not significant.

## Data Availability

The original contributions presented in this study are included in the article/Supplementary Materials. Further inquiries can be directed to the corresponding author.

## Supplementary Materials

The following supporting information can be downloaded at: https://www.mdpi.com/article/10.3390/microorganisms14102140/s1, Figure S1: Relationship between LDH release and MTT viability; Figure S2: Myxothiazol-sensitive component of MitoSOX fluorescence.
