# Supplementary material for: Quercetin Attenuates Streptococcus pneumoniae-Induced Oxidative Stress, Inflammatory Mediator Release, and Cytotoxicity in Human Bronchial Epithelial Cells: An In Vitro Study Using an Acapsular Strain
Source: Microorganisms. 2026 Sep 23;14(10):2140. doi: 10.3390/microorganisms14102140 (PMC13707923; doi:10.3390/microorganisms14102140)

Supplement:

Supplementary figure: S1

Relationship between LDH release and MTT viability. Group mean LDH release (% cytotoxicity) was plotted against MTT metabolic viability (% of control) for control, *S. pneumoniae*, *S. pneumoniae* + quercetin, and quercetin-only groups. Error bars represent the corresponding group variability. LDH release was strongly inversely correlated with MTT viability (Pearson $r = -0.89$, 95% CI −0.95 to −0.78, $p < 0.001$), supporting concordance between membrane injury and metabolic viability readouts.

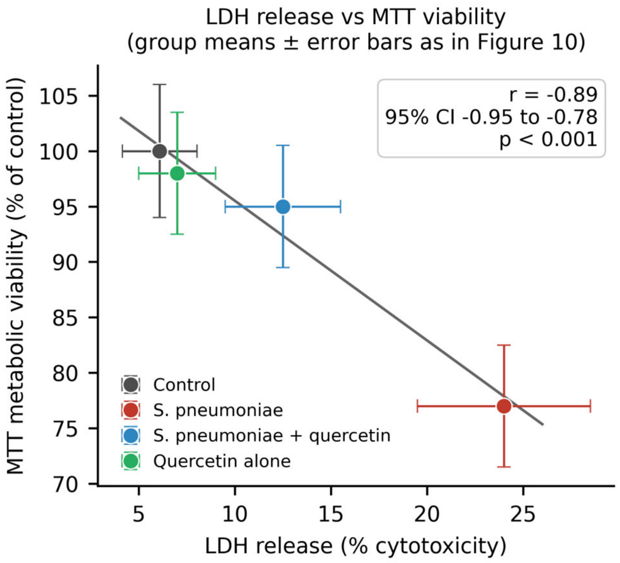

Supplementary figure: S2

Myxothiazol-sensitive component of MitoSOX fluorescence. MitoSOX fluorescence was measured in control, *S. pneumoniae*-exposed, and *S. pneumoniae* + quercetin groups in the presence or absence of the mitochondrial complex III inhibitor myxothiazol (1 μM). Myxothiazol markedly reduced MitoSOX fluorescence across all conditions, supporting a substantial mitochondrial electron transport-dependent contribution to the measured signal. Data are presented as mean ± SEM with individual biological replicates shown; n = 6 per condition.

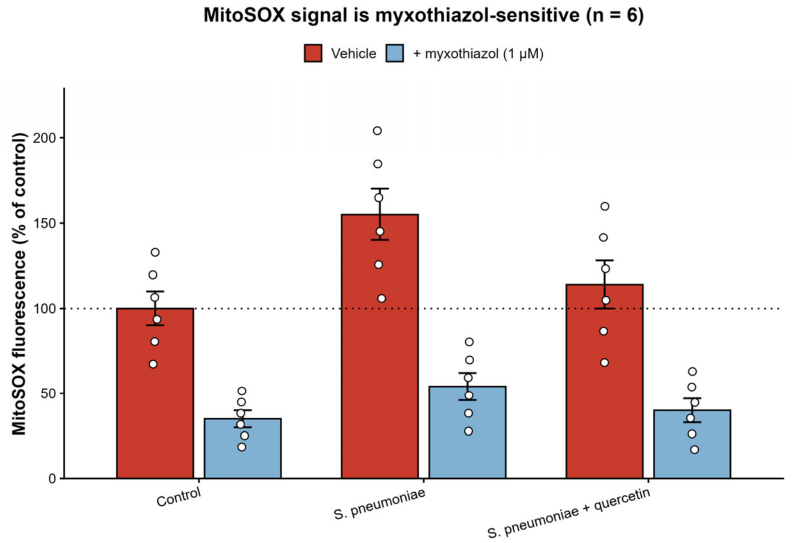

Supplement: Supplementary file 1 [file microorganisms-14-02140-s001.zip › microorganisms-4444408-supplementary.pdf]
